# Exoskeletons and orthoses: classification, design challenges and future directions

**DOI:** 10.1186/1743-0003-6-21

**Published:** 2009-06-18

**Authors:** Hugh Herr

**Affiliations:** 1 MIT Media Lab, Massachusetts Institute of Technology, 20 Ames Street, Room 424, Cambridge, Massachusetts, USA; 2 MIT-Harvard Division of Health Science and Technology, Massachusetts Institute of Technology, 20 Ames Street, Room 424, Cambridge, Massachusetts, USA

## Abstract

For over a century, technologists and scientists have actively sought the development of exoskeletons and orthoses designed to augment human economy, strength, and endurance. While there are still many challenges associated with exoskeletal and orthotic design that have yet to be perfected, the advances in the field have been truly impressive. In this commentary, I first classify exoskeletons and orthoses into devices that act in series and in parallel to a human limb, providing a few examples within each category. This classification is then followed by a discussion of major design challenges and future research directions critical to the field of exoskeletons and orthoses.

## Introduction

The current series of the Journal of NeuroEngineering and Rehabilitation (JNER) is dedicated to recent advances in robotic exoskeletons and powered orthoses. The articles in this special issue cover a broad spectrum of embodiments, from orthotic devices to assist individuals suffering from limb pathology to limb exoskeletons designed to augment normal, intact limb function.

To set the stage for this special issue, I classify exoskeletons and orthoses into four categories and provide design examples within each of these. I discuss devices that act in series with a human limb to increase limb length and displacement, and devices that act in parallel with a human limb to increase human locomotory economy, augment joint strength, and increase endurance or strength. For each exoskeletal type, I provide a design overview of hardware, actuation, sensory, and control systems for a few characteristic devices that have been described in the literature, and when available, describe the results of any quantitative evaluation of the effectiveness of the devices in performing their intended tasks. Finally, I end with a discussion of the major design challenges that have yet to be overcome, and possible future directions that may provide resolutions to these design difficulties.

For the purposes of this commentary, exoskeletons and orthoses are defined as mechanical devices that are essentially anthropomorphic in nature, are 'worn' by an operator and fit closely to the body, and work in concert with the operator's movements. In general, the term 'exoskeleton' is used to describe a device that augments the performance of an able-bodied wearer, whereas the term 'orthosis' is typically used to describe a device that is used to assist a person with a limb pathology.

It is perhaps worth noting that the term "exoskeleton" has come to describe systems that are comprised of more than just a passive protective and supporting shell, as its usage in biology would suggest. "Exoskeleton" within our research community is taken to include mechanical structures, as well as associated actuators, visco-elastic components, sensors and control elements.

## Series-limb exoskeletons

Elastic elements in the body, such as ligaments and tendons, have long been known to play a critical role in the economy and stability of movement [1-7]. Humans and other animals use these tissues to reduce impact losses while storing substantial quantities of energy when striking the ground, and to provide propulsion during terminal stance in walking, running and jumping. Such biological strategies have inspired designers of running track surfaces and wearable devices such as shoes and exoskeletons.

**Figure 1:**
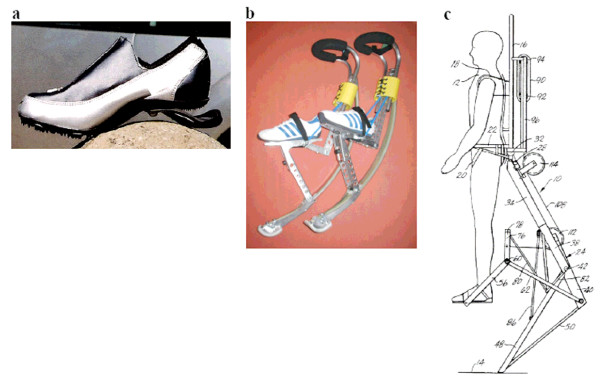
**Shoes and exoskeletons that act in series with the human lower limb**. Examples are the Springbuck shoe [10,11], the PowerSkip exoskeleton , and the SpringWalker exoskeleton [12] shown in 1a, 1b, and 1c, respectively.

Previous studies have shown that a compliant running track can improve performance by increasing running speed by a few percent and may also reduce the risk of injury [8]. In another study on elastic running surfaces, the authors found a range of compliant ground surface stiffnesses that improved metabolic running economy [9]. Similarly, previous studies have shown that wearable mechanisms in series with the biological leg can reduce the metabolic cost of running by lowering impact losses and by providing energy return. A running shoe called the Springbuck, designed with a carbon composite elastic midsole, was shown to improve shock absorption and metabolic economy at moderate running speeds (see Figure 1a); [10,11]. Although metabolic economy improved when runners used this elastic shoe rather than a conventional shoe design without an elastic midsole, the advantage was found to be modest (~2%). Elastic exoskeletons in series with the human leg have been developed that store and release far greater strain energy than the running track surface of [8] or the Springbuck shoe [10,11] (~5 Joules/step for track and shoe versus ~80 Joules/step for elastic exoskeletons), and therefore it was believed that such exoskeletons would augment human running speed and economy. Notable inventions in this exoskeletal class are the PowerSkip and the SpringWalker shown in Figure 1b and 1c, respectively ; [12]. However, although these devices clearly augment jumping height, they have not been shown to improve peak running speed nor running economy. In fact, in a study conducted by the U.S. Army Research Institute of Environmental Medicine (ARIEM) in Natick, Massachusetts, the SpringWalker *increased *metabolic cost by 20% compared to locomotion without the device [Personal Communication: Peter Frykman]. For this study, mass was added to the subject's back equal to the SpringWalker mass.

### Parallel-limb exoskeletons for load transfer

Here we discuss exoskeletons that act in parallel with the human lower limb for load transfer to the ground. Perhaps an in-series leg exoskeleton like the SpringWalker (Figure 1c) increases the metabolic cost of running because the limb length of the human plus machine is substantially increased, thereby increasing both the work at the hip to protract the leg during the aerial phase and the overall energetic demand to stabilize movement, overcoming any potential advantage of extending limb length. Additionally, with an in-series leg exoskeleton device, the ground reaction forces are still borne by the human leg. In contrast, with a parallel mechanism, body weight could be transferred through the exoskeleton directly to the ground, decreasing the loads borne by the biological limbs and lowering the metabolic demands to walk, run, and hop. Furthermore, such a parallel exoskeleton would not increase limb length, thereby not increasing the overall energetic demand to stabilize movement.

**Figure 2:**
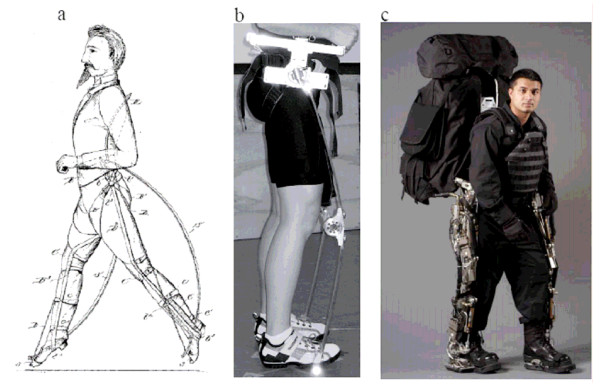
**Exoskeletons that act in parallel with the human lower limb for load transfer to the ground**. Examples are Yagn's running aid [14], MIT's hopping exoskeleton [15,16], and Kazerooni's load-carrying exoskeleton [18,19] shown in 2a, 2b, and 2c, respectively.

The earliest mention of such a parallel exoskeleton is a set of United States patents granted in 1890 to Nicholas Yagn [13,14]. His invention, shown in Figure 2a, comprises long leaf springs operating in parallel to the legs, and was intended to augment the running abilities of the Russian Army. Each leg spring was designed to engage at foot strike to effectively transfer the body's weight to the ground and to reduce the forces borne by the stance leg during each running stance period. During the aerial phase, the parallel leg spring was designed to disengage in order to allow the biological leg to freely flex and to enable the foot to clear the ground. Although Yagn's mechanism was designed to augment running, there is no record that the device was ever built and successfully demonstrated.

The MIT Biomechatronics Group recently built an elastic exoskeleton similar to Yagn's design. However, its intended application was not for running augmentation, but for lowering the metabolic demands of continuous hopping [15,16]. The exoskeleton, shown in Figure 2b, comprises fiberglass leaf springs that span the entire leg, and is capable of transferring body weight directly to the ground during the stance period. In distinction to Yagn's exoskeleton, the MIT device does not include a clutch to disengage the exoskeletal leaf spring during the aerial phase since such a clutching control was deemed unnecessary for hopping. Without accounting for the added weight of each exoskeleton, wearing the exoskeleton reduced net metabolic power for continuous hopping by an average of 24% compared to normal hopping [16]. When hoppers utilized external parallel springs, they decreased the mechanical work performed by the legs and substantially reduced metabolic demand compared to hopping without wearing an exoskeleton. Since the biomechanics of hopping are similar to that of running, it seems plausible that the effects of wearing an exoskeleton during hopping could predict the biomechanical and metabolic effects of wearing an exoskeleton during running, and that substantial energetic advantages might be achieved while running with a highly elastic, parallel leg exoskeleton. Clearly, for the goal of augmenting human running performance, lightweight and highly elastic leg exoskeletons that act in parallel with the human leg provide a research area of critical importance.

Parallel-limb exoskeletons have also been advanced to augment the load-carrying capacity of humans [17-32]. This type of leg exoskeleton could benefit people who engage in load carrying by increasing load capacity, lessening the likelihood of leg or back injury, improving metabolic locomotory economy, and/or reducing the perceived level of difficulty. One such exoskeletal design is shown in Figure 2c, or the Berkeley Lower Extremity Exoskeleton (BLEEX) developed by Professor Kazerooni. One of the distinguishing features of this exoskeleton is that it is energetically autonomous, or carries its own power source. Indeed, its developers claim it as the first "load-bearing and energetically autonomous" exoskeleton [17].

BLEEX features three degrees of freedom (DOF) at the hip, one at the knee, and three at the ankle. Of these, four are actuated: hip flexion/extension, hip abduction/adduction, knee flexion/extension, and ankle flexion/extension. Of the non-actuated joints, the ankle inversion/eversion and hip rotation joints are spring-loaded, and the ankle rotation joint is free-spinning [18]. The kinematics and actuation requirements of the exoskeleton were designed by assuming behavior similar to that of a 75 kg human and utilizing clinical gait analysis data for walking [18,19].

Interesting features of the kinematic design of the exoskeleton include a hip "rotation" joint that is shared between the two legs of the exoskeleton, and therefore, does not intersect with the wearer's hip joints. Similarly, the inversion/eversion joint at the ankle is not co-located with the human joint, but is set to the lateral side of the foot for simplicity. The other five rotational DOF's of the exoskeleton coincide with the joints of the wearer [18].

The exoskeleton is actuated via bidirectional linear hydraulic cylinders mounted in a triangular configuration with the rotary joints, resulting in an effective moment arm that varies with joint angle. BLEEX consumes an average of 1143 Watts of hydraulic power during level-ground walking, as well as 200 Watts of electrical power for the electronics and control. In contrast, a similarly sized, 75 kg human consumes approximately 165 W of metabolic power during level-ground walking [18,19].

BLEEX was designed with linear hydraulic actuators since they were the "smallest actuation option available" based on their "high specific power (ratio of actuator power to actuator weight)" [18]. However, a further study determined that electric motor actuation significantly decreased power consumption during level walking in comparison to hydraulic actuation [20]. The weight of the implementation of the electrically-actuated joint, however, was approximately twice that of their hydraulically-actuated joint (4.1 kg vs. 2.1 kg).

The control scheme of the BLEEX seeks to *minimize *the use of sensory information from the human/exoskeleton interaction, and instead, utilizes mainly sensory information from the exoskeleton. Similarly to a bipedal robot, the exoskeleton can balance on its own, but the human wearer must provide a forward guiding force to direct the system during walking. The control system utilizes the information from eight encoders and sixteen linear accelerometers to determine angle, angular velocity, and angular acceleration of each of the eight actuated joints, a foot switch, and load distribution sensor per foot to determine ground contact and force distribution between the feet during double stance, eight single-axis force sensors for use in force control of each of the actuators, and an inclinometer to determine the orientation of the backpack with respect to gravity [18].

In order to achieve their goal of being energetically autonomous with such an actuator selection, significant effort was invested in developing a hybrid hydraulic-electric portable power supply [21].

In terms of performance, users wearing BLEEX can reportedly support a load of up to 75 kg while walking at 0.9 m/s, and can walk at speeds of up to 1.3 m/s without the load. A second generation of the Berkeley exoskeleton is currently in testing. The new device is approximately half the weight of the original exoskeleton (~14 kg [22]), in part due to the implementation of electric actuation with a hydraulic transmission system. A laboratory spin-off company called Berkeley Bionics (Berkeley, CA) has been formed in order to market the exoskeleton technology.

## Parallel-limb exoskeletons for torque and work augmentation

Here we discuss exoskeletons that act in parallel with the human joint(s) for torque and work augmentation. Many parallel-limb exoskeletons have been developed to augment joint torque and work [33-58]. In distinction to the load-carrying exoskeletons mentioned in the last section, this type of exoskeletal and orthotic device does not transfer substantial load to the ground, but simply augments joint torque and work. This type of leg exoskeleton could improve walking and running metabolic economy, or might be used to reduce joint pain or increase joint strength in paralyzed or weak joints.

**Figure 3:**
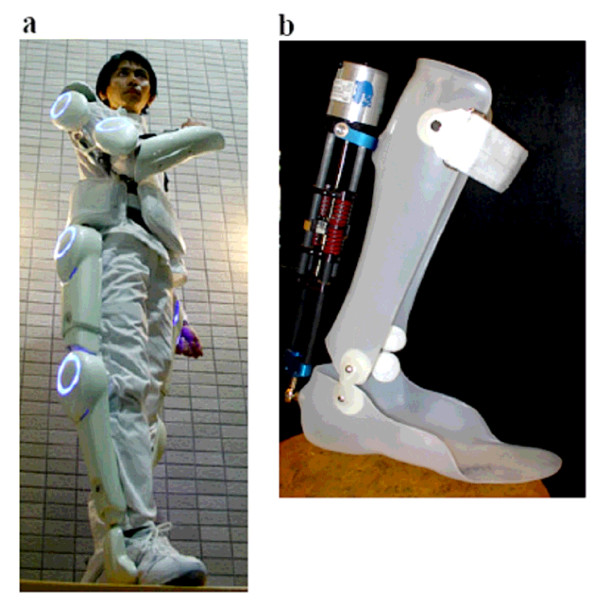
**Exoskeletons that act in parallel with human joint(s) for torque and work augmentation**. Examples are the HAL 5 exoskeleton [49,50] and the MIT active ankle-foot orthosis [52] shown in 3a and 3b, respectively.

One such exoskeletal design is shown in Figure 3a. At the University of Tsukuba in Japan, Professor Yoshiyuki Sankai and his team have been developing an exoskeleton concept that is targeted for both performance-augmenting and rehabilitative purposes [49,50]. The leg structure of the full-body HAL-5 exoskeleton powers the flexion/extension joints at the hip and knee via a DC motor with harmonic drive placed directly on the joints. The ankle flexion/extension degree of freedom is passive. The lower-limb components interface with the wearer via a number of connections: a special shoe with ground reaction force sensors, harnesses on the calf and thigh, and a wide waist belt.

The HAL-5 system utilizes a number of sensing modalities for control: skin-surface EMG electrodes placed below the hip and above the knee on both the anterior (front) and posterior (back) sides of the wearer's body, potentiometers for joint angle measurement, ground reaction force sensors, a gyroscope and accelerometer mounted on the backpack for torso posture estimation. These sensing modalities are used in two control systems that together determine user intent and operate the suit: an EMG-based system and a walking pattern-based system. Reportedly, it takes two months to optimally calibrate the exoskeleton for a specific user [22].

HAL-5 is currently in the process of being readied for commercialization. Modifications from previous versions include upper-body limbs, lighter and more compact power units, longer battery life (approximately 160 minutes continuous operating time), and a more cosmetic shell. The total weight of the full-body device is 21 kg. Cyberdyne (Tsukuba, Japan, ), a company spun off from Sankai's lab, is responsible for the commercialization of the product.

The ability of HAL to improve performance by increasing the user's capacity to lift and press large loads has been demonstrated . An operator wearing HAL can lift up to 40 kg more than they can manage unaided. Additionally, the device increases the user's 'leg press' capability from 100 to 180 kg. However, to date no peer-reviewed, quantitative results have been published highlighting the effectiveness of the exoskeleton's lower-limb components for the improvement of locomotory function.

A second example of a parallel-limb orthosis that augments joint torque and work is shown in Figure 3b. The MIT Biomechatronics Group developed a powered ankle-foot orthosis [52] to assist drop-foot gait, a deficit affecting many persons who have experienced a stroke, or with multiple sclerosis or cerebral palsy, among others. The device consists of a modified passive ankle-foot orthosis with the addition of a series elastic actuator (SEA) that is controlled based on ground force and angle sensory information. Using the SEA, the device varies the impedance of the ankle during controlled plantar flexion in stance, and assists with dorsiflexion during the swing phase of walking.

In clinical trials, the MIT active ankle-foot orthosis (AFO) was shown to improve the gait of drop-foot patients by increasing walking speed, reducing the instances of "foot slap", creating better symmetry with the unaffected leg, and providing assistance during powered plantar flexion. Subjects' feedback was also favorable. The AFO is relatively compact and consumes a small amount of power (10W average electrical power consumption), and current work at iWalk, LLC , a spin-off company from MIT, is focused on developing an energetically autonomous, portable version of the device.

### Parallel-limb exoskeletons that increase human endurance

Throughout the human body hundreds of muscles exert forces to stiffen and move the limbs and torso. During exhaustive exercise, only a small portion of these muscles fatigue. For a repetitive anaerobic activity, a parallel-limb exoskeleton could be designed to redistribute the cyclic work load over a greater number of muscles for the purpose of delaying the onset of fatigue. In such a strategy, springs within the exoskeleton could be stretched by muscles that would not normally fatigue if the exercise were conducted without the mechanism. The energy stored by the exoskeleton could then be used to assist those muscles that would typically fatigue, possibly improving endurance capacity.

**Figure 4:**
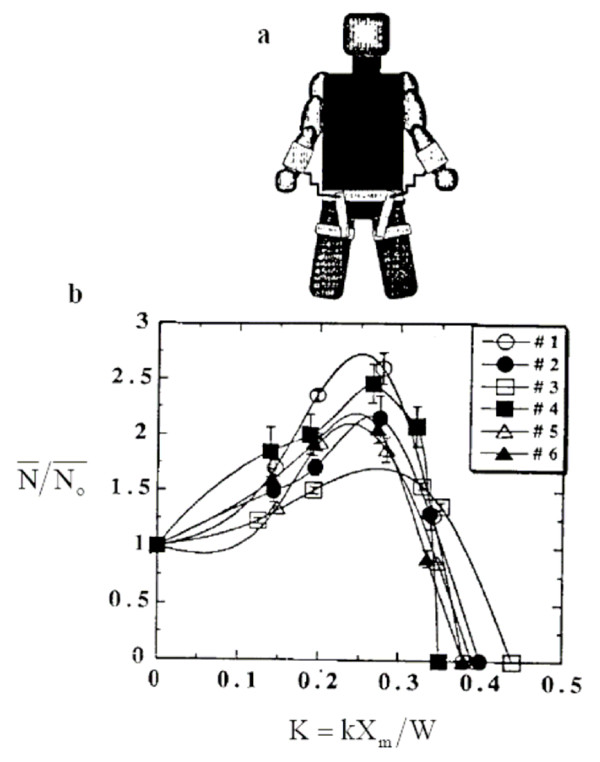
**Exoskeletons that act in parallel with a human limb for endurance augmentation**. An example is the MIT climbing exoskeleton [59] shown in 4a. As shown in 4b, when the stiffness of the mechanism was optimally tuned, endurance was increased from 1.5-fold to 2.5-fold across the six human subjects evaluated. The mean number of cycles to exhaustion (), or the endurance, normalized by the mean value at zero stiffness (), is plotted in Fig. 4b versus the dimensionless arm spring stiffness (K). K is defined as the measured stiffness of the added spring (k) multiplied by the maximum distance the spring was stretched (X_m_), and divided by the subject's body weight (W). For each subject, a cubic spline curve passes through the mean of the normalized cycle values (± SE) at each of the five stiffness values. Endurance is maximized around K ~0.25 for each subject.

To test whether it is indeed possible for an exoskeleton to amplify endurance using this strategy, researchers [59] conducted an experiment on six human subjects each wearing a simple exoskeleton comprised of two springs that connected each wrist to a waist harness (see Figure 4a). The springs were in equilibrium when both elbows were fully flexed with the wrists positioned at chest height. With this mechanism, a subject performed the following cyclic activity until complete exhaustion using a given spring stiffness. From a sitting position, a subject fully extended his arms to grasp a pull-up bar directly overhead, stretching the arm springs. With the assistance of the stretched springs, the subject lifted his body upwards with his arms until his chin cleared the bar. Then the subject stood on the seat of a chair, released the bar, and sat down on the chair. Note that the cycle did not include lowering the body with the arms after pulling up. Using this approach, energy was only stored in the springs by extending the arms upward. Each subject performed the experiment five times with a given spring stiffness using a total of five different spring stiffnesses. The order in which spring stiffnesses were used was randomized to rule out any sequential effects. In addition, each subject was required to use the same time to sit down after pulling up so that the time in which the arms were not being used during each cycle did not change. Between experiments, a subject was given two to three days of rest.

The experimental results are shown in Figure 4b. The endurance was maximized around K ~0.25 for each subject. Further, the endurance with an exoskeleton increased by 1.5-fold to 2.5-fold compared to the endurance when no exoskeleton assist was employed. Using a mathematical model of the human arm and exoskeleton, researchers [59] related overall muscle efficiency to exoskeletal stiffness. The model predicted that muscle efficiency was maximized at the same dimensionless stiffness where endurance reached its maximum (K~0.25 in Figure 4b), suggesting that the endurance changes were a consequence of changes in the efficiency with which the body performed the required work for each cycle.

There are many applications for this class of exoskeleton. For example, a crutch was constructed with an orthotic elbow spring to maximize the endurance of physically-challenged persons in climbing stairs and slopes [60]. When the crutch user flexes both elbows to place the crutch tips onto the next stair tread, orthotic elbow springs compress and store energy. This stored energy then assists the crutch user during elbow extension, helping to lift the body up the next step, and delaying the onset of bicep and tricep muscle fatigue. In future developments, robotic exoskeletons and powered orthoses could be put forth that actively vary impedance to optimally redistribute the body's work load over a greater muscle volume, maximizing the efficiency with which the body is able to perform mechanical work and significantly augmenting human endurance.

## Design challenges and future directions

Although great progress has been made in the century-long effort to design and implement robotic exoskeletons and powered orthoses, many design challenges still remain. Remarkably, a portable leg exoskeleton has yet to be developed that demonstrates a significant decrease in the metabolic demands of walking or running. Many complicated devices have been developed that *increase *consumption, such as the SpringWalker [12] and the MIT load-carrying exoskeleton [27-29].

There are many factors that continue to limit the performance of exoskeletons and orthoses. Today's powered devices are often heavy with limited torque and power, making the wearer's movements difficult to augment. Current devices are often both unnatural in shape and noisy, factors that negatively influence device cosmesis. Given current limitations in actuator technology, continued research and development in artificial muscle actuators is of critical importance to the field of wearable devices. Electroactive polymers have shown considerable promise as artificial muscles, but technical challenges still remain for their implementation [61,62]. These challenges include improving the actuator's durability and lifetime at high levels of performance, scaling up the actuator size to meet the force and stroke needs of exoskeletal/orthotic devices, and advancing efficient and compact driving electronics. Although difficulties remain, electroactive polymer muscles may offer considerable advantages to wearable robotic devices, allowing for integrated joint impedance and motive force controllability, noise-free operation, and anthropomorphic device morphologies. An improved understanding of muscle and tendon function during human movement tasks may shed light on how artificial muscles should ideally attach to the exoskeletal frame (monoarticular vs. polyarticular actuation) and be controlled to produce enhanced biomimetic limb dynamics. For example, neuromechanical models that capture the major features of human walking (e.g. [63,64]) may improve understanding of musculoskeletal morphology and neural control and lead to analogous improvements in the design of economical, stable and low-mass exoskeletons for human walking augmentation.

Another factor limiting today's exoskeletons and orthoses is the lack of direct information exchange between the human wearer's nervous system and the wearable device. Continued advancements in neural technology will be of critical importance to the field of wearable robotics. Peripheral sensors placed inside muscle to measure the electromyographic signal, or centrally-placed sensors into the motor cortex, may be used to assess motor intent by future exoskeletal control systems [65,66]. Neural implants may have the potential to be used for sensory feedback to the nerves or brain, thus allowing the exoskeletal wearer to have some form of kinetic and kinematic sensory information from the wearable device [67].

Current exoskeletal/orthotic devices are also limited by their mechanical interface. Today's interface designs often cause discomfort to the wearer, limiting the length of time that a device can be worn. It is certainly an achievable goal to provide comfortable and effective mechanical interfaces with the human body. Contemporary external prosthetic limbs attach to the human body most commonly via a prosthetic socket that is custom fabricated to an individual's own contours and anatomical needs. Although not a perfectly comfortable interface, today's prosthetic sockets nonetheless allow amputee athletes to run marathons, compete in the Ironman Triathlons, and even climb Mount Everest. One strategy employed in the fabrication of modern prostheses is to digitize the surface of the residual limb, creating a three dimensional digital description of the residual limb contours. Once the amputee's limb has been scanned, their geometric data are sent to a computer aided manufacturing (CAM) facility where a new prosthetic socket is fabricated rapidly and at relatively low cost.

In the future such file-to-factory rapid processes may be employed for the design and construction of exoskeletal and orthotic devices. In this framework, a three dimensional scanning procedure would produce a digital record of the human body's outer shape. This geometric data along with other anatomical information, such as data on tissue compliance and anatomically-sensitive areas, would be combined with strength and endurance information from a physical fitness diagnostic examination. Such anatomical and fitness data, combined with the wearer's augmentation requirements, would provide an individual's design specification profile. An exoskeleton, customized to fit the wearer's outer anatomical features and physiological demands, would then be designed as a 'second skin'. Such a skin would be made compliant in body regions having bony protuberances, and more rigid in areas of high tissue compliance. The exoskeletal skin would be so intimate with the human body that external shear forces applied to the exoskeleton would not produce relative movement between the exoskeletal inner surface and the wearer's own skin, eliminating skin sores resulting from device rubbing. Compliant artificial muscles, sensors, electronics and power supply would be embedded within the three dimensional construct, offering full protection of these components from environmental disturbances such as dust and moisture. Once designed, device construction would unite additive and subtractive fabrication processes to deposit materials with varied properties (stiffness and density variations) across the entire exoskeletal volume using large scale 3-D printers and robotic arms.

## Exoskeletons and the future of mobility

During the 20^th ^century, investments in human-mobility technology primarily focused on wheeled devices. Relatively little investment was focused on the advancement of anthropomorphic exoskeletal technologies that allow humans to move bipedally at enhanced speeds and with reduced effort and metabolic cost. It seems likely that in the 21st century more investments will be made to drive innovation in this important area. The fact that large automobile companies, such as Honda and Toyota, have recently begun exoskeletal research programs is an indication of this technological shift. Perhaps in the latter half of this century, exoskeletons and orthoses will be as pervasive in society as wheeled vehicles are today. That would allow the elderly, the physically challenged and persons with normal intact physiologies to achieve a level of mobility not yet achieved. That would be a day in which the automobile – that large, metal box with four wheels – is replaced with wearable, all-terrain exoskeletal devices, allowing city streets to be transformed from 20^th ^century pavement to dirt, trees and rocks. One can only hope.

## Competing interests

The author is founder of iWalk, LLC, a company dedicated to the commercialization of wearable robotic technology for human augmentation.
